# Examining the association between prenatal and perinatal adversity and the psychotic experiences in childhood

**DOI:** 10.1017/S0033291724000187

**Published:** 2024-07

**Authors:** Lorna Staines, Niamh Dooley, Colm Healy, Ian Kelleher, David Cotter, Mary Cannon

**Affiliations:** 1 Department of Psychiatry, Royal College of Surgeons in Ireland, Dublin 2, Ireland; 2 Division of Psychiatry, Centre for Clinical Brain Sciences, University of Edinburgh, Edinburgh EH10 5HF, UK; 3 Department of Psychiatry, Beaumont Hospital, Dublin 9, Ireland

**Keywords:** persistent psychotic experiences, prenatal complications, psychosis, psychotic experiences

## Abstract

**Background:**

Prenatal and perinatal complications are established risk factors for psychotic disorder, but far less is known about these measures and psychotic experiences (PEs). We investigated the longitudinal effect of prenatal risk factors (maternal behavior, medication complications) and perinatal risk factors (birth weight, medical complications) on frequency of PEs. We also examined the cumulative risk of prenatal/perinatal risk factors, and differences between transient PE, persistent PE, and controls.

**Methods:**

The Adolescent Brain Cognitive Development study is a large child cohort (age 9–10 at baseline; *n* = 11 872 with PE data). PEs were measured longitudinally using the Prodromal Questionnaire-Brief, Child version, and included only if reported as distressing. Mixed-effects models were used for analysis, controlling for random effects, and a substantial number of fixed-effects covariates.

**Results:**

Urinary tract infection (*β* = 0.11, 95% confidence interval [CI] 0.03–0.19) and severe anemia (*β* = 0.18, 95% CI 0.07–0.29) increased frequency of distressing PEs in childhood. Number of prenatal complications increased frequency of PEs (*β* = 0.03, 95% CI 0.01–0.06) and risk of persistent PEs (odds ratio [OR] = 1.08, 95% CI 1.01–1.15). Maternal smoking was associated with an increased frequency of PEs (*β* = 0.11, 95% CI 0.04–0.18) and persistent PEs (OR = 1.31, 95% CI 1.04–1.66). Maternal substance use was a risk factor for a 48% increased risk of persistent PEs (OR = 1.48, 95% CI 1.08–2.01). Perinatal complications showed no effect on PEs.

**Conclusions:**

This study provides evidence that certain prenatal medical complications (severe nausea, severe anemia), cumulative number of prenatal medical complications, and maternal behaviors (smoking during pregnancy), increased frequency of distressing PEs in childhood. Maternal smoking and substance use, as well as cumulative number of prenatal complications increased risk of persistent PEs.

## Introduction

Psychosis continuum proposes psychotic experiences (PEs) and psychotic disorder exist on a continuum and have a shared etiology (van Os, 2003; van Os, Linscott, Myin-Germeys, Delespaul, & Krabbendam, 2009). PEs are defined as infrequent hallucinations or delusions occurring in the absence of a psychotic disorder (Staines et al., 2022). The presence of PEs, particularly persistent and/or distressing PE, is associated with poorer functional and occupational outcomes, higher healthcare needs, and an elevated risk of developing a mental disorder (Bhavsar, McGuire, MacCabe, Oliver, & Fusar-Poli, 2018; Healy et al., 2019; Jeppesen et al., 2015; Rimvall et al., 2020a, 2020b, 2020c; Staines et al., 2023a).

Prenatal and perinatal risk factors have been robustly shown to have a small-to-moderate effect on rates of psychotic disorder (Cannon, Jones, & Murray, 2002; Davies et al., 2020). PE research has focused primarily on examining maternal behaviors such as smoking, alcohol consumption, and substance use. There is evidence that smoking during pregnancy is linked to increased risk for PEs (Barkhuizen, Taylor, Freeman, & Ronald, 2019; Betts, Williams, Najman, Scott, & Alati, 2014; Dorrington et al., 2014; Zammit et al., 2009b). There is inconsistent evidence for alcohol consumption (Staines et al., 2022), with some papers finding it has an effect, and others not finding an association (Gregersen, Dreier, & Strandberg-Larsen, 2020; Zammit et al., 2009a). The evidence of the association between maternal cannabis use and later PEs in children is more consistent, indicating maternal cannabis use increases risk of PEs in children (Bogdan, Sarah, & Alexander, 2022; Bourque, Afzali, O'Leary-Barrett, & Conrod, 2017; Fine et al., 2019; Paul et al., 2020).

Other pre/perinatal complications, such as medical complications, have been less frequently examined. Two previous cohort studies (Betts et al., 2014; Zammit et al., 2009a) have measured other prenatal and perinatal complications as risk factors for PEs. Zammit et al. (2009a) found evidence that infection during pregnancy, any resuscitation after birth, and a low Apgar score, were all associated with an increased risk for PEs in childhood. Betts et al. (2014) found evidence that stressful life events during pregnancy increased risk of PEs in adulthood, but no evidence of any medical prenatal/perinatal complication. There is inconsistent evidence about the effects of birthweight, some studies finding that higher birthweight was protective (Thomas et al., 2009), and lower birthweight increased risk (Drakesmith et al., 2016), while others have found no effect of birthweight (Betts et al., 2014).

One area that has not been investigated to date is the cumulative effect of prenatal and perinatal complications. Pre- and perinatal complications often co-occur (Bryson, Ioannou, Rulyak, & Critchlow, 2003; Östlund, Haglund, & Hanson, 2004; Yogev, Xenakis, & Langer, 2004), and the cumulative effect of prenatal complications have been shown to increase psychopathology in childhood (Roffman et al., 2021; Sood et al., 2001). Additionally, previous studies (Betts et al., 2014; Zammit et al., 2009a) have only examined the relationship between prenatal and perinatal complications with PEs at a single time-point. To our knowledge, no study has investigated whether these effects are developmentally specific or enduring. Similarly, no study has distinguished if these risk factors can differentiate those with multiple PE events (persistent), compared to one off PE events (transient).

The current study aims to answer three key questions:

1. Are pre- and perinatal risk factors associated with frequency of PEs across late childhood (age 9–12)?
2. Is there a cumulative effect of prenatal/perinatal risk factors on frequency of PEs?
3. Are prenatal/perinatal risk factors differently related to transient and persistent PEs?

## Methods

### Participants

This study used data from the Adolescent Brain Cognitive Development (ABCD) study (abcdstudy.org), an ongoing prospective cohort study; full details are available elsewhere (Jernigan & Brown, 2018; Karcher et al., 2018). Briefly, children were recruited at age 9–10 at baseline (*n* = 11 875) and followed up every 1–2 years. The ABCD sample was designed to mirror US population demographics by recruiting through geographically, demographically, and socioeconomically diverse school systems surrounding each of the research sites (Garavan et al., 2018), with the exception that ABCD at baseline oversampled for non-singleton births (Garavan et al., 2018). Exclusion criteria include non-fluency in English (child/parent), or if the child had contraindications to magnetic resonance imaging, extreme prematurity (<28 weeks gestation), history of neurological disorders, current diagnosis of schizophrenia, substance-use disorder, intellectual disability, or moderate/severe autism spectrum disorder. All measures used were from baseline (age 9–10), time-point 1 (age 10–11), and time-point 2 (age 11–12).

Within study sites, consenting parents and assenting children were primarily recruited through a probability sample of schools, summer camp programs, and community volunteers. The University of California at San Diego (San Diego, CA, USA) Institutional Review Board was responsible for the ethical oversight of the ABCD study. The secondary analysis of the data was approved by the Research Ethics Committee for the Royal College of Surgeons in Ireland.

### Measures

Data were collected using in-person interviews. Parent report used the primary caregiver, including biological mothers (85%), biological fathers (10%), adoptive parents (2.5%), custodial parents (1%), and ‘other’ (1.5%).

### Exposures

Prenatal and perinatal complications were collected using the primary caregiver report at baseline using the ABCD Developmental History Questionnaire (Kessler et al., 2009; Merikangas, Avenevoli, Costello, Koretz, & Kessler, 2009). In line with previous research, these were scored dichotomously (absent/present) and analyzed collectively to measure cumulative risk (Roffman et al., 2021).

#### Prenatal complications

The following prenatal measures were included. These were collected at baseline from the primary caregiver, reported as dichotomous measure (Yes/No). The measures included were: severe nausea beyond 6th month of pregnancy, prenatal diabetes, high blood pressure, persistent proteinuria, urinary tract infection (UTI), pre-eclampsia/eclampsia/toxemia, severe anemia, rubella in the first 3 months of pregnancy, severe gallbladder attack, placental issues (previa, abruption, other), excessive bleeding requiring treatment, accident requiring medical attention, or other complication requiring medical attention. Each prenatal complication was examined separately, and the total number of prenatal complications was computed (0–12) for the prenatal complications cumulative score.

#### Perinatal complications

Perinatal complications were collected from the primary caregiver, reported as a binary response (Yes/No). The following prenatal measures were included, reported as dichotomous measures: being born jaundiced, requiring oxygen at birth, born blue, with a slow heartbeat, not breathing at first, rhesus incompatibility, a blood transfusion, or convulsions. Each perinatal complication was examined separately, and the total number of delivery complications was computed (0–8) for the cumulative perinatal complications score.

#### Fetal growth

In line with previous research (Class, Rickert, Larsson, Lichtenstein, & D'Onofrio, 2014; Dooley et al., 2022) we opted to use fetal growth, rather than birthweight, by calculating the appropriateness of weight for their prenatal age. Measures of prenatal age (in weeks) and birth weight (kg) were parent reported at baseline. To calculate fetal growth, we used a linear regression to obtain the residuals from the association between prenatal age and birthweight. This allowed us to estimate the variance in birthweight, accounting for the child's prenatal age, giving us a proxy estimate of fetal growth.

#### Maternal behavior

Maternal smoking, alcohol consumption, and substance use was collected from the Developmental History Questionnaire (Kessler et al., 2009; Merikangas et al., 2009) and was defined as engaging in those activities at any point during pregnancy. Maternal smoking was collected from question ‘tobacco? How many times a day?’, treated as a binary if they smoked during pregnancy, regardless of frequency. Alcohol consumption was measured as ‘Alcohol? Average drinks per week?’, treated as a binary if they drank during pregnancy, regardless of frequency. Substance use was created by combining questions of ‘Marijuana? How many times per day?/Cocaine/Crack? How many times per day?/Heroin/Morphine? How many times per day?/Oxycontin? How many times per day?’. A binary ‘yes/no’ variable was created for substance use, based on a positive answer to any of the substance use questions, regardless of frequency.

### Covariates

A demographic questionnaire measured child ethnicity and sex, and parental income. Ethnicity was measured as a categorical variable (Black, White, Asian, Hispanic, other), and sex (male/female), both reported at baseline. Income level was calculated as a categorical variable of combined (if two-parent household) income (<$5k, $5–11k, $12k–15 999, $16k–24,999, $25k–34 999, $35k–49 999, $50k–74 999, $75k–99 999, $100k–199 999, $200k+). Maternal age at time of birth was reported by the mother at baseline.

Family history of mental illness was collected using the parent-reported Family History Assessment (Rice et al., 1995). We calculated the number of family members with any mental illness (alcohol addiction, drug addiction, conduct disorder, depression, bipolar, anxiety, psychosis, suicide attempt/death by suicide, hospitalization for mental illness, use of clinical services). A second measure examining specifically the number of relatives with psychosis was also calculated.

## Outcomes

### Psychotic experiences

PEs were measured using the self-report Prodromal Questionnaire-Brief, Child version (PQ-BC) (Karcher et al., 2018). This is adapted from the adult version (Loewy, Pearson, Vinogradov, Bearden, & Cannon, 2011), and similarly measures 21 items on a range of positive psychotic symptoms. For each symptom the participant endorsed, they were asked whether the symptom ‘bothered them (Yes/No)’. Those who affirmatively reported that the PE was distressing were asked to score level of distress (1–5).

PE was measured as frequency of distressing PE, defined as a participant scoring distress about PE as ⩾3, at each time-point respectively. In the PQ-BC, distress is rated on a 5 point scale, using appropriate age visuals to help children assess distress; for full details see Karcher et al. (2018). The cut-off of ⩾3 is in line with previous research (Karcher et al., 2022). Inclusion of this cut-off was used to ensure that an actual PE is being reported, utilizing previous studies which identify distress as a marker of a significant PE in children (Rimvall et al., 2020a, 2020c).

### Persistent *v.* transient PEs

Persistent was defined as at least one distressing PE at more than one time (McGrath et al., 2015). Transience was defined as a distressing PE at only one time-point. Control was defined as reporting no distressing PE at any time-point. Only those with data at all 3 time-points were included for this analysis.

## Analysis

Mixed models were used for each model; the random effects allowed us to capture variance across research sites and families. In all models, continuous variables were standardized. To allow for standardization, income was treated as a continuous variable. To examine if the risk factors were independently associated with distressing childhood PEs, the model was adjusted for a number of known risk factors for PEs. The fully adjusted model accounted for demographic and sample differences in sex, ethnicity, over-sampling of twins/triplets, maternal age, socioeconomic status, family history of psychosis/mental illness, and maternal behaviors (smoking, alcohol consumption, and substance use).

For the analysis of individual prenatal/perinatal risk factors, and for the cumulative risk of pre-/peri-natal risk factors, Gamma distribution linear mixed-effects models were used. The main effect, and the interaction between the exposure (prenatal/perinatal complication) and time are also reported. This allowed us to assess whether the effects of exposures remitted as the participants got older, or remained consistent.

To account for multiple comparisons for the individual prenatal/perinatal risk factors the Benjamini and Hochberg's (1995) false-discovery date correction for multiple comparisons was used. This was used for the main effect and interactions, respectively.

Each maternal behavior was also examined as a prenatal risk factor. For these models, all measures expect maternal behaviors were included as covariates. To account for multiple comparisons the Benjamini and Hochberg's (1995) false-discovery date correction for multiple comparisons was also used. Examining differences between transient and persistent PEs, binomial distribution regression logistic mixed-effects models were used.

Participants without the outcomes, exposures, or confounders were excluded from the respective analyses where data were missing. The included sample, for each model, is reported in each table.

## Results

### Descriptive

At baseline, 11 872 completed the PQ-BC; those who had not completed baseline (*n* = 4) were excluded from analysis. At follow-up T1, 651 did not complete the measure (*n* = 11 221), and at follow-up T2 1462 did not complete the measure (*n* = 10 410). We found small-to-moderate correlations between prenatal and perinatal complications (online Supplementary eFigures 1 and 2). Children who reported PE at baseline were compared to controls (Table 1).

**Table 1.** Differences in demographic, income, familial history, maternal risk behaviors, prenatal complications, perinatal complications, and fetal growth between children with PEs and controls, at baseline

	PEs	Controls	χ^2^ /*r*	p-value
*N* (%)	3158 (26.8)	8714 (73.2)		
Sex, *n* (% female)	1532 (48.51)	4147 (47.6)	χ^2^ = 0.77	0.38
Age in years, *m* (s.d.)	9.85 (0.61)	9.95 (0.62)	*r* = 0.04***	<0.001
Multiple perinatal, *n* (%)	561 (23.9)	1528 (22.1)	χ^2^ = 2.95	0.09
Ethnicity, *n*				
White	1292 (41.0)	4880 (56.1)	χ^2^ = 211.66***	<0.001
Black	654 (20.7)	1125 (35.7)	χ^2^ = 109.93***	<0.001
Hispanic	805 (25.5)	1601 (50.8)	χ^2^ = 72.12***	<0.001
Asian	46 (1.5)	206 (6.5)	χ^2^ = 8.77**	0.003
Other	357 (11.3)	888 (28.2)	χ^2^ = 2.93	0.09
Income level, *n* (%)				
<$5000	155 (4.9)	261 (8.3)	χ^2^ = 26.05***	<0.001
$5000–11 000	147 (4.7)	275 (8.7)	χ^2^ = 15.91***	<0.001
$12 000–15 999	96 (3.0)	177 (5.6)	χ^2^ = 10.80**	0.001
$16 000–24 999	206 (6.5)	317 (10.1)	χ^2^ = 47.59***	<0.001
$25 000–34 999	215 (6.8)	438 (13.9)	χ^2^ = 15.21***	<0.001
$35 000–49 999	298 (9.4)	636 (20.2)	χ^2^ = 16.06***	<0.001
$50 000–74 999	448 (14.2)	1051 (33.3)	χ^2^ = 11.12**	0.001
$75 000–99 999	400 (12.7)	1171 (37.1)	χ^2^ = 0.66	0.42
$100 000–199 999	670 (21.2)	2645 (83.9)	χ^2^ = 91.70***	<0.001
$200 000+	223 (7.1)	1025 (32.5)	χ^2^ = 51.57***	<0.001
Maternal age in years, *m* (s.d.)	28.43 (6.45)	29.75 (6.17)	*r* = 0.09***	
No. of family members with history of mental disorders, *m* (s.d.)	2.87 (2.70)	2.77 (2.62)	*r* = 0.01	0.12
No. of family members with history of psychosis, *m* (s.d.)	0.12 (0.41)	0.09 (0.36)	*r* = 0.04***	<0.001
Gestational complications, *n* (%)				
Severe nausea after 6 months	529 (17.43)	1092 (12.93)	χ^2^ = 36.98***	<0.001
High blood pressure	347 (11.43)	804 (9.54)	χ^2^ = 8.59**	0.003
UTI	285 (9.43)	586 (6.99)	χ^2^ =18.35***	<0.001
Persistent proteinuria	12 (0.4)	46 (0.55)	χ^2^ = 0.72	0.40
Excessive bleeding requiring treatment	156 (5.11)	365 (4.30)	χ^2^ = 3.24	0.07
Pre-eclampsia/eclampsia/toxemia	247 (8.15)	628 (7.43)	χ^2^ = 1.55	0.21
Severe anemia	179 (5.88)	318 (3.76)	χ^2^ = 23.78***	<0.001
Rubella in the first 3 months of pregnancy	3 (0.1)	15 (0.18)	χ^2^ = 0.46	0.50
Severe gallbladder attack	41 (1.34)	90 (1.06)	χ^2^ = 1.36	0.25
Placental issues (previa, abruption, other)	76 (2.49)	272 (3.21)	χ^2^ = 3.72	0.054
Prenatal diabetes	263 (8.65)	552 (3.21)	χ^2^ = 15.06***	<0.001
Accident requiring medical attention	56 (1.83)	156 (1.84)	χ^2^ = 0.00	0.99
Other complication requiring medical attention	259 (8.49)	724 (8.54)	χ^2^ = 0.00	0.95
Prenatal issues *N*, *m* (s.d.)	0.798 (1.11)	0.662 (1.07)	*r* = 0.06***	<0.001
Maternal smoking, *n* (%)	520 (15.6)	1048 (12.3)	χ^2^ = 42.86***	<0.001
Maternal alcohol consumption, *n* (%)	713 (24.2)	2115 (25.9)	χ^2^ = 3.13	0.08
Maternal substance use, *n* (%)	299 (9.6)	544 (6.3)	χ^2^ = 36.55***	<0.001
Birth complications				
Born jaundiced	510 (16.63)	1387 (16.29)	χ^2^ = 0.16	0.69
Requiring oxygen at birth	301 (9.85)	820 (9.63)	χ^2^ = 0.20	0.75
Born blue	105 (3.44)	262 (3.08)	χ^2^ = 0.79	0.37
Born with a slow heartbeat	95 (3.11)	227 (2.68)	χ^2^ = 1.39	0.24
Born not breathing at first	150 (4.91)	394 (4.63)	χ^2^ = 0.35	0.56
Rhesus incompatibility	76 (2.51)	210 (2.49)	χ^2^ = 0.00	0.99
Requiring a blood transfusion	14 (0.45)	42 (0.49)	χ^2^ = 0.01	0.91
Convulsions at birth	9 (0.29)	9 (0.11)	χ^2^ = 3.96*	0.046
Perinatal complications *N*, *m* (s.d.)	0.399 (0.80)	0.385 (0.77)	*r* = 0.00	0.73
Fetal growth, *m* (s.d.)	−0.04 (0.68)	0.015 (0.66)	*r* = 0.04***	<0.001

PE, psychotic experience (distressing); χ^2^, chi-squared test; *r*, effect size of Wilcoxon test.

***p < 0.001, **p< 0.01, *p < 0.05.

Children whose parents reported their ethnicity as Black, Hispanic, or Asian were proportionally more likely to report PE than those identified as White (Table 1). Income level showed a trend of more people having PEs being in the lower income brackets, and less people with PE in the higher income brackets (Table 1). Maternal age was significantly younger for those with PE compared to controls (Table 1). Number of family members with mental disorder/psychosis was also higher for PE compared to controls (Table 1).

Considering group-level differences of the main exposures, maternal smoking and substance use were higher in those with PE than controls at baseline. Several prenatal medical complications and one perinatal complication were more common in those with PE at baseline (Table 1). The number of prenatal complications, but not perinatal complications, were higher in those with PE at baseline (Table 1). Fetal growth was on average below average in the PE group, while it was above average in the control group (Table 1).

Any prenatal/perinatal measure reported by less than 100 people with PEs was excluded from the main analysis. Prenatal complications excluded on this criteria included persistent proteinuria, rubella in the first 3 months of pregnancy, a severe gallbladder attack, placental issues, or accident requiring medical attention (Table 1). Perinatal complications excluded were blood transfusions or convulsions (Table 1).

### Prenatal and perinatal risk factors for PE

Two included individual measures of prenatal complication measures (UTI and severe anemia) were associated with an increase in frequency of PEs in childhood, which survived correction for multiple comparisons (Table 2). Maternal smoking had a significant main effect (Table 2) when correcting for multiple comparisons, showing maternal smoking increases the frequency of distressing PEs in children. As children entered adolescence the main effect of maternal smoking was moderately attenuated (Table 2).

**Table 2.** Prenatal risk factors on rates of PEs in childhood, accounting for demographic, economic, familial history, and maternal risk behaviors

		Main effect			T1 interaction with exposure			T2 interaction with exposure		
Measure	*n*	*β* (s.e.)	95% CI	*t*	△*β* (s.e.)	95% CI	*t*	△*β* (s.e.)	95% CI	*t*
Medical complications										
Severe nausea after 6 months	22 600	0.07 (0.03)	0.01–0.14	2.35	−0.02 (0.03)	−0.09 to 0.04	−0.78	−0.06 (0.03)	−0.12 to 0.00	−1.83
High blood pressure	22 534	0.01 (0.04)	−0.05 to 0.08	0.40	−0.00 (0.03)	−0.07 to 0.06	−0.13	−0.02 (0.03)	−0.09 to 0.04	−0.69
UTI	22 429	0.11 (0.04)	0.03–0.19	2.58*	−0.01 (0.04)	−0.09 to 0.08	−0.14	−0.06 (0.04)	−0.14 to 0.03	−1.36
Excessive bleeding requiring treatment	22 649	0.07 (0.05)	−0.03 to 0.17	1.42	−0.04 (0.05)	−0.13 to 0.06	−0.80	−0.01 (0.05)	−0.10 to 0.09	−0.11
Pre-eclampsia/eclampsia/toxemia	22 556	0.06 (0.04)	−0.01 to 0.14	1.61	−0.04 (0.04)	−0.11 to 0.04	−0.91	−0.05 (0.04)	−0.13 to 0.03	−1.32
Severe anemia	22 596	0.18 (0.06)	0.07–0.29	3.05*	−0.08 (0.06)	−0.20 to 0.04	−1.31	−0.15 (0.06)	−0.26 to −0.03	−2.52
Prenatal diabetes	22 571	0.08 (0.04)	0.00–0.16	1.99	−0.07 (0.04)	−0.15 to 0.01	−1.70	−0.07 (0.04)	−0.15 to 0.02	−1.58
Other complication requiring medical attention	22 622	0.03 (0.04)	−0.04 to 0.10	0.95	−0.01 (0.04)	−0.08 to 0.06	−0.36	−0.02 (0.03)	−0.09 to 0.05	−0.48
Maternal behavior										
Maternal smoking^a^	23 728	0.11 (0.03)	0.04–0.18	3.24**	−0.06 (0.03)	−0.13 to 0.01	−1.79	−0.08 (0.04)	−0.15 to −0.01	−2.38*
Maternal alcohol consumption^a^	22 808	−0.00 (0.02)	−0.05 to 0.05	−0.03	0.001 (0.02)	−0.04 to 0.05	0.06	−0.00 (0.02)	−0.05 to 0.04	−0.19
Maternal substance use^a^	23 864	0.10 (0.05)	0.00–0.19	2.00	−0.00 (0.05)	−0.10 to 0.10	−0.05	−0.01 (0.05)	−0.11 to 0.09	−0.24

PE, distressing psychotic experiences; *β*, estimate; △*β*=, change in estimate; *t*, *t*-value.

*p*-Value is corrected for multiple comparisons, using Benjamini and Hochberg's (1995) false-discovery rate correction.

a Maternal smoking, alcohol consumption, and substance use did not do the fully adjusted model, only controlling for minimally adjusted model, family income, family history of psychosis, and mental disorders; model fully adjusted; accounting for demographic (sex, multiple births, ethnicity, maternal age and random effect of site, and family ID), family income, family history of psychosis and mental disorder, maternal risk behaviors in pregnancy (maternal alcohol consumption, smoking, substance use).

**p < 0.01, *p < 0.05.

Fetal growth was not associated with frequency of PEs in childhood (Table 3). No perinatal medical complication was associated with an elevated risk for PE in childhood (Table 3).

**Table 3.** Perinatal risk factors on rates of PEs in childhood, accounting for demographic, economic, familial history, and maternal risk behaviors

		Main effect			T1 interaction with exposure			T2 interaction with exposure		
Measure	*n*	*β* (s.e.)	95% CI	*t*	△*β* (s.e.)	95% CI	*t*	△*β* (s.e.)	95% CI	*t*
Medical complications										
Born jaundiced	22 560	−0.01 (0.03)	−0.06– 0.05	−0.25	−0.00 (0.03)	−0.06 to 0.05	−0.14	−0.01 (0.03)	−0.06 to 0.05	−0.25
Requiring oxygen at birth	22 542	0.02 (0.03)	−0.05 to 0.08	0.54	0.02 (0.04)	−0.05 to 0.08	0.58	−0.03 (0.03)	−0.10 to 0.03	−0.96
Born blue	22 485	0.05 (0.06)	−0.06 to 0.17	0.95	−0.01 (0.06)	−0.13 to 0.10	−0.23	−0.01 (0.06)	−0.12 to 0.11	−0.14
Born with a slow heartbeat	22 449	−0.01 (0.06)	−0.13 to 0.12	−0.11	0.07 (0.07)	−0.06 to 0.20	1.06	0.00 (0.07)	−0.13 to 0.13	0.04
Born not breathing at first	22 533	−0.01 (0.05)	−0.10 to 0.09	−0.12	−0.00 (0.05)	−0.10 to 0.10	−0.02	−0.04 (0.05)	−0.14 to 0.05	−0.91
Rhesus incompatibility	22 408	−0.01 (0.06)	−0.13 to 0.11	−0.14	0.05 (0.06)	−0.06 to 0.17	0.90	0.08 (0.06)	−0.04 to 0.17	1.26
Birth weight										
Fetal growth	23 313	−0.03 (0.02)	−0.07 to 0.00	−1.86	0.02 (0.02)	−0.01 to 0.05	1.79	0.03 (0.02)	0.00 to 0.06	2.02

PE, distressing psychotic experiences.

Model fully adjusted; accounting for demographic (sex, multiple births, ethnicity, maternal age and random effect of site, and family ID), family income, family history of psychosis and mental disorder, maternal risk behaviors in pregnancy (maternal alcohol consumption, smoking, substance use).

*p*-value is corrected for multiple comparisons, using Benjamini and Hochberg's (1995) false-discovery rate correction.

**p < 0.01, *p < 0.05.

### Cumulative risk of prenatal/perinatal risk factors and PE

**Figure 1.**
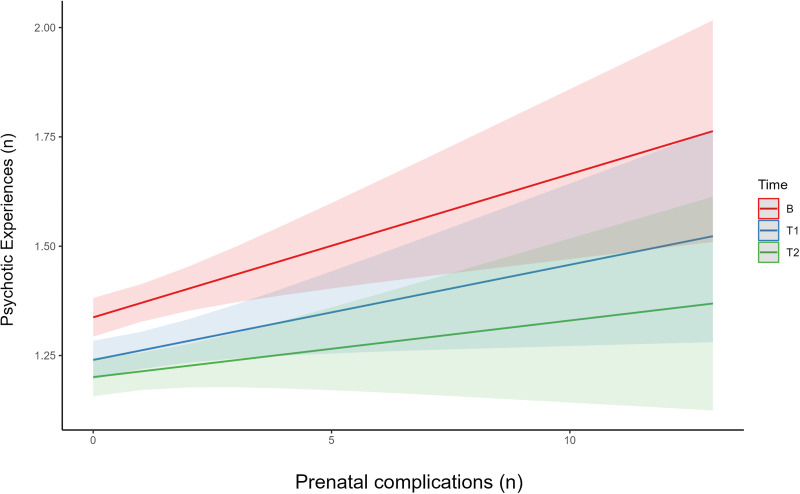
Interaction effects between number of prenatal complications on number of PE, divided by time-point. The interaction graph allows prediction of the effect of number of prenatal complications on rates of PE, split into the three time-points (baseline, follow-up 1, and 2). This graph also allows us to examine if there in a difference in effect at different time-points, based on the slope of the line. This graph indicates that while there is an effect of number of prenatal complications on rate of PE, it declines at later time-points i.e. as the children get older. B, baseline time-point, T1, follow-up 1 (1 year), T2, follow up 2 (2 years).

Cumulative risk of prenatal complications (median = 0; interquartile range [IQR] = 1; range(0–13)), and perinatal complications (median = 0; IQR = 1; range(0–8)) was low. Controlling for covariates, every additional prenatal complication was associated with a small increase in frequency of PEs (*β* = 0.03; 95% confidence interval [CI] 0.01–0.05, *t* = 3.12, *p* = 0.002). This effect showed a non-significant reduction at time-point 1 (*β* = −0.01; 95% CI −0.03 to 0.01, *t* = −1.00, *p* = 0.32), and moderately attenuated by time-point 2, i.e. as the child enters adolescence (*β* = −0.02; 95% CI −0.04 to −0.00], *t* = −2.00, *p* = 0.05) (Fig. 1). The cumulative risk of perinatal risk factors was non-significant (*β* = 0.00; 95% CI −0.03 to 0.01], *t* = 0.29, *p* = 0.82).

### Persistent *v*. transient PEs

Only those with measures of PEs at all three time-points were included for analysis (*n* = 10 182). Participants were grouped into control (*n* = 6261), transient (*n* = 2236), and persistent (*n* = 1685).

Persistent and transient PE groups had a significantly higher number of prenatal complications in the fully adjusted model, compared to the control group (Table 4). For each prenatal complication, participants were 8% more likely to be in the persistent or transient group, compared to controls (Table 4). There were no significant differences in prenatal complications between the persistent PE and transient PE groups. The number of perinatal complications and average fetal growth were not associated with group membership (Table 4).

**Table 4.** Effects of prenatal complications, perinatal complications, fetal growth on risk of PEs, comparing between different PE groups (control, transient, persistent)

	Persistent *v*. transient			Persistent *v*. control			Transient *v*. control		
	*n*	Odds ratio (OR)	95% CI	*n*	OR	95% CI	*n*	OR	95% CI
No. of prenatal complications	2490	1.00	0.93–108	5531	1.08*	1.01–1.15	5971	1.08*	1.02–1.14
No. of perinatal complications	2495	1.00	0.91–1.11	5538	1.04	0.94–1.14	5977	1.04	0.96–1.12
Fetal growth	2427	1.01	0.87–1.18	5416	0.97	0.84–1.13	5841	0.97	0.86–1.09
Maternal smoking^a^	2607	1.05	0.82–1.33	5787	1.31*	1.04–1.66	6230	1.32**	1.08–1.61
Maternal alcohol consumption^a^	2505	0.99	0.82–1.21	5556	1.04	0.86–1.24	5995	1.05	0.91–1.22
Maternal substance use^a^	2628	1.32	0.94–1.83	5815	1.48**	1.08–2.01	6257	1.11	0.83–1.48

Persistent, reported distressing PE at 2–3 time-points; transient, reported distressing PE at only 1 time-point; control, reported distressing PE at no time-points; *β*, estimate; *E*, standard error; *t*, *t*-value.

a Maternal smoking, alcohol consumption, and substance use did not do the fully adjusted model, only controlling for minimally adjusted model, family income, family history of psychosis and mental disorders. Model 1: accounting for demographic (sex, multiple births, ethnicity, maternal age and random effect of site, and family ID). Model 2: accounting for family income, family history of psychosis and mental disorder, maternal risk behaviors in pregnancy (maternal alcohol consumption, smoking, substance use) + model 1.

***p < 0.001, **p < 0.01, *p < 0.05.

In the fully adjusted model, maternal smoking was a group differentiator for persistent and transient PEs compared to control, representing a 31% and 32% risk respectively (Table 4). Maternal substance use was a differentiator for persistent PE compared to control, representing an elevated risk of being in the persistent PE group by 48% (Table 4). No measure of maternal behavior was a differentiator between the transient and persistent PE groups (Table 4).

## Discussion

This study examined prenatal and perinatal factors as risk factors for frequency of distressing childhood PE and persistent PE, and found several risk factors; UTIs, severe anemia, cumulative risk of prenatal medication complication, maternal smoking, and maternal substance use.

Previous research has shown that a UTI during pregnancy is associated with preterm labor (Balachandran et al., 2022), susceptibility to pediatric infections (Cohen, Gutvirtz, Wainstock, & Sheiner, 2019), and symptoms of attention deficit and hyperactivity disorder (ADHD) (Mann & McDermott, 2011). Our finding adds to this literature by showing the presence of maternal UTI increases the frequency of distressing PE across childhood. Similarly, severe anemia has previously been linked to higher rates of perinatal complications (Shi et al., 2022) and neurodevelopmental difficulties in offspring, including ADHD and autism spectrum disorder (Wiegersma, Dalman, Lee, Karlsson, & Gardner, 2019).

One explanation for why prenatal complications could increase risk for subsequent PEs is the role of inflammation. Inflammation is a natural biological response to infection or physical trauma (Scott, Khan, Cook, & Duronio, 2004). Evidence suggests that inflammation (acute and chronic) is associated with psychotic phenomena (Föcking et al., 2019; Mongan et al., 2021; Mongan, Ramesar, Föcking, Cannon, & Cotter, 2020). It has been hypothesized that aberrant inflammatory processes may be the explanation for this process (Mongan et al., 2020). Within this context, maternal infection may be the mechanism by which aberrant inflammatory processes develop, through abnormal immune responses in utero (Anderson et al., 2016; Khandaker, Zimbron, Lewis, & Jones, 2013). Previous studies which have examined maternal acute inflammation during pregnancy but did not find an association with PEs (Anderson et al., 2016; Ramsay et al., 2021). Chronic inflammation is a more significant inflammatory marker for subsequent psychotic symptoms (Byrne et al., 2022; Rasmussen et al., 2016). Prenatal complications in this study may be experienced as chronic/long lasting e.g. UTI. Therefore, the finding of this study may support the role of chronic inflammation. However, to determine the mechanisms behind the finding, additional research using biological data is required.

Maternal smoking was a risk factor for frequency and persistent distressing PE in this study. Previous research in PE has observed maternal smoking as a risk factor for PE (Barkhuizen et al., 2019; Dorrington et al., 2014; Zammit et al., 2009a). As was observed in this study, maternal smoking as a risk factor declined in adolescence. Maternal substance use was also found to be a risk factor for PE. Previous research has primarily focused on maternal cannabis consumption, with consistent evidence showing it increases rates of PE (Bogdan et al., 2022; Bolhuis et al., 2018; Fine et al., 2019; Paul et al., 2020; Staines et al., 2023b). Explanations for the association between substance use and mental disorders have been suggested to be due to a shared etiology with addictive behaviors (Myles et al., 2012). Similarly, addiction shows shared environmental risk factors to mental disorders and PE e.g. socioeconomic status (Patrick, Wightman, Schoeni, & Schulenberg, 2012; Staines et al., 2022; Werner, Malaspina, & Rabinowitz, 2007). It is therefore possible that the associations found in this study may be driven by shared genetic and/or environmental factors between the pregnant person and infant.

Second, smoking has been associated with chronic inflammation (Lee, Taneja, & Vassallo, 2012), and it is possible in utero exposure to smoking might also be explained by the same mechanism discussed above. One consideration is that cannabis consumption is often combined with tobacco use (Weinberger et al., 2019). In the ABCD study, of the participants exposed to cannabis usage during pregnancy (*n* = 655), 44% (*n* = 289) were also exposed to tobacco use during pregnancy (Paul et al., 2020). Therefore, it should not be ruled out that some of the effects observed in this study may have been driven in part by those who smoked tobacco and cannabis in combination. However, this group only represents 18%, of all those who smoked tobacco while pregnant (Table 1). Overall this study suggests that tobacco and substance use represent an independent risk for distressing childhood PEs.

Perinatal complications did not show a significant effect in this study, differing from previous research on PEs (Betts et al., 2014; Zammit et al., 2009a). One consideration is that prenatal complications in this study are experienced over extended periods of time or chronically, while exposure to perinatal complications is becoming shorter in newer cohorts. Evidence shows that countries with substantial health care services have lower rates of birth asphyxia and hypoxia (Mukhtar-Yola et al., 2018; World Health Organization, 2004). Previous cohorts on this topic were born in 1981 (Betts et al., 2014) and 1991–1992 (Zammit et al., 2009a), in comparison to the ABCD sample born in 2008 (Garavan et al., 2018).

Finally, lower than average fetal growth in this study was not found to be a risk factor for PEs, or higher than average fetal growth as a protective factor. Previous studies (Cannon et al., 2002; Davies et al., 2020) have observed an association between lower birth weight and psychotic disorder. The PE literature has been less conclusive, with mixed evidence for birthweight (Betts et al., 2014; Drakesmith et al., 2016) and some evidence for birthweight as a protective factor (Thomas et al., 2009).

## Limitations

First, all pre- and perinatal information was reported retrospectively by the primary caregiver 9–10 years post-partum. Therefore, it cannot be assumed that all medical complications or maternal behaviors were reported accurately. Similarly, 95% of the records were collected from the biological parents (85% mother, 10% father). This means that 15% of the total ABCD sample were not reported by the individuals who were pregnant, so risk of missing data or recall bias may be more likely in this subgroup. Second, there are some limitations to the generalizability of the ABCD sample used, specifically with the over representation of participants in the higher income bracket ($100 000–200 000). This could be a sample with better access to healthcare than the general population. Additionally, current diagnosis of schizophrenia or substance use in the child was an exclusion criterion, meaning it may not represent the most severe end of the psychosis spectrum. PEs were self-report from relatively young participants, whose interpretation of the question is unknown (age 9–10 baseline, 11–12 time-point 2). Distress level was included in PE ratings in an attempt to distinguish strong perceptual experiences from common everyday occurrences. Finally, this study controlled for a significant number of known covariates, to consider if the prenatal/perinatal risk factors were independent risk factors for childhood distressing PEs. This was an advantage of the large sample size, and addressed limitations of previous studies. However, several covariates e.g. family history of mental disorders, can also increase risk for prenatal complications (Sūdžiūtė et al., 2020), as well as being a risk factor for PEs. Therefore, in considering prenatal complications, it is important to be cognizant that these risk factors may not independently occur, and so improving prenatal care is only part of reducing these risk factors.

## Conclusions

Prenatal and perinatal complications have been described as the ‘canaries in the coalmine’ (Cannon, Healy, Clarke, & Cotter, 2020) for psychosis, and this research supports that description. Most substantially, maternal behaviors (smoking, substance use) showed the largest effect, and highlight the continued need for education and promotion of healthy behaviors during pregnancy, and support for treatable complications of pregnancy, such as severe anemia. The interaction between time and prenatal complications suggests that these may be predominantly risk factors for childhood PEs. However, PEs in childhood can have long-term adverse outcomes (Rimvall et al., 2020a, 2020b). Therefore, prevention of maternal infection should be a priority, not just for the health of the pregnant person, but also for the future health of the fetus.

## Supporting information

Staines et al. supplementary material Staines et al. supplementary material

## Supplementary material

The supplementary material for this article can be found at https://doi.org/10.1017/S0033291724000187.
