# Supplementary material for: Examining the association between prenatal and perinatal adversity and the psychotic experiences in childhood
Source: Psychol Med. 2024 Mar 4;54(9):2087–98. doi: 10.1017/S0033291724000187 (PMC11413340; doi:10.1017/S0033291724000187)
Supplement: Staines et al. supplementary material [file S0033291724000187sup001.docx]

Supplementary material

[Appendix 1. Correlation between prenatal complications 2](#_Toc132127915)

[Appendix 2. Correlation between perinatal complications 3](#_Toc132127916)

# eFigure 1. Correlation between prenatal complications


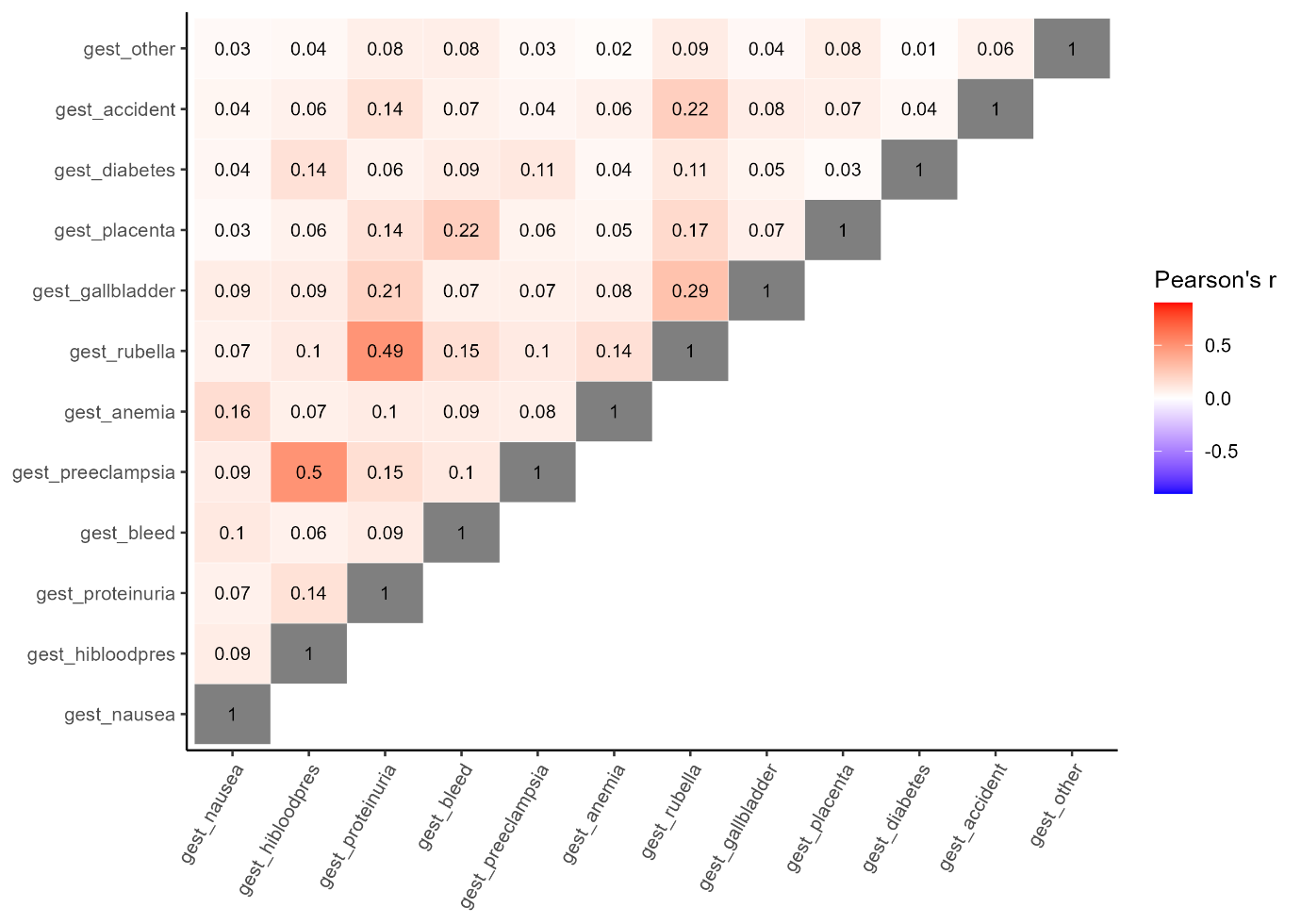


Gest_nausea= severe nausea after 6 months of pregnancy, gest_highbloodpres = High blood pressure, gest_proteinuria = persistent proteinuria, gest_bleed = bleeding requiring treatment, gest_preeclampisa = pre-eclampsia/eclampsia/toxaemia, gest_anemia = severe anemia, gest_rubella = rubella in the first three months of pregnancy, gest_gallbladder = Severe gallbladder attack, gest_placenta = , placental issues (previa, abruption, other), gest_diabetes = gestational diabetes, gest_accident = accident requiring medical attention, gest_other = other complication requiring medical attention.

# eFigure 2. Correlation between perinatal complications


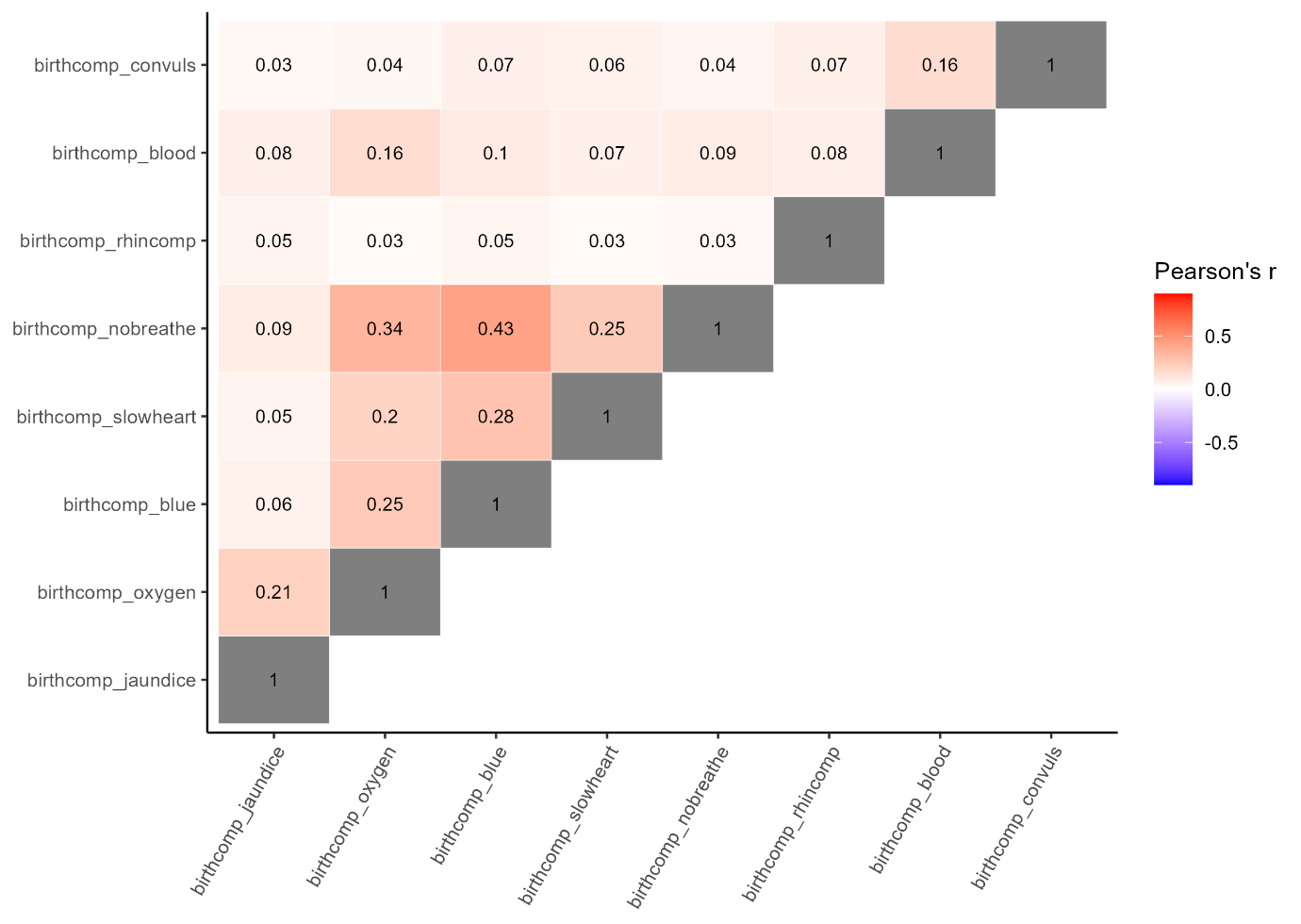


Birthcomp_jaundice = being born jaundiced, birthcomp_oxygen = requiring oxygen at birth, birthcomp_blue = born blue, birthcomp_slowheart = with a slow heartbeat, birthcomp_nobreathe = not breathing at first, birthcomp_rhinocomp = rhesus incompatibility, birthcomp_blood = a blood transfusion, birthcomp_convuls = convulsions.
